# An In Vitro Comparison of Shear Bond Strength for Heated Composite Resin With Three Conventional Luting Cements

**DOI:** 10.7759/cureus.47110

**Published:** 2023-10-16

**Authors:** Prasad Aravind, Abhinav Mohan Kallat, Prem Kumar Sivabalan, Shibi Mathew Velurethu, Nirosha Vijayan, Cimmy Augustine

**Affiliations:** 1 Department of Prosthodontics, Crown and Bridge, Mahe Institute of Dental Sciences and Hospital, Puducherry, IND; 2 Department of Prosthodontics, Crown and Bridge, Pushpagiri College of Dental Sciences, Thiruvalla, IND

**Keywords:** dental restorations, luting cements, composite resin, lithium di silicate, shear bond strength

## Abstract

Background*:* This research set out to collate and contrast three popular luting agents-heated composite resin, resin-modified glass ionomer cement (RMGIC), and resin cement, and light-cure resin cement by measuring their shear bond strengths. Shear bond strength was measured between lithium disilicate discs (IPS E-max) and specimens luted with heated composite resin (Tetric N-Ceram, Ivoclar Vivadent), self-adhesive resin cement (3M ESPE Rely X U200), light-activated resin cement (Rely X Veneer cement), and resin-modified glass ionomer cement (Fuji Plus, GC America). A comparison was made between the shear bond strength of standard luting cement and heated composite resin on lithium disilicate discs.

Materials and methods: Forty-eight lithium disilicate disc samples are collected and put on acrylic blocks for this investigation. To improve luting cement adhesion, the discs are etched with 5% hydrofluoric acid (HF) gel. For easier handling and lower viscosity during luting, the composite resin is heated to between 55 and 68°C on a digital wax melter. Shear bond strength tests were executed with the universal testing device after the following luting cement was applied in the center of the test specimen (lithium disilicate discs). Statistics software was used for the calculations and analysis.

Results: In accordance with the findings of the tests, shear bond strengths ranged from 2.2851 ± 0.5901 for nanohybrid composite resin to 7.3740 ± 0.6969 for self-adhesive resin cement and 4.4647 ± 0.9774 for light-activated resin cement. A statistically significant (p≤0.001) difference between the groups was found. Mean shear bond strength was significantly highest in the self-adhesive resin cement group, followed by the light-activated resin cement group, resin-modified GIC, and least with the nanohybrid composite resin group.

Conclusion: Composite resins; in fixation of indirect restorations can have their viscosity reduced by preheating in a device, but they must be employed as soon as possible after removal. Standardizing the methods of heating composite resins for cementation is necessary to achieve desirable outcomes and direct the physician in their application. Although preheating composite resins for luting operations can be utilized to decrease the material's viscosity and enhance the restoration setting; it may not increase bond strength.

## Introduction

All-ceramic restorations have grown in popularity, not long ago. The need for dental restorations, namely ceramic inlays, onlays, veneers, and full crowns, has improved. As a result, more and more all-ceramic restorations are being bonded with composite resin cements for added durability [1,2]. Composite resin cements, of which there are many varieties, are made up of an adhesive and a composite resin made of polymerizing particle filler [3-5].

Short-span fixed partial dentures (FPDs) may be fabricated from a variety of ceramics, including a lithium disilicate glass-ceramic core veneered with a sintered glass-ceramic (IPS Empress 2; Ivoclar Vivadent, Schaan, Liechtenstein) [6,7]. Clinical achievement and lifetime of prostheses made from the various dental ceramics used today [8] are influenced by a number of aspects, like luting cement and the cementation method [9].

Dental composite resins can be pre-heated or pre-warmed using the novel, easy, and unique thermal-assisted light polymerization technology. For optimal characteristics in restorations [10] and to keep the interface intact [11,12], appropriate polymerization of dental composite is required. Preheat the composite resin before placement for thermal-assisted light polymerization [13-15] to increase the rate of conversion of composite and reduce resistance to flow, thereby enhancing marginal modification of the cavity. Pre-heating has become an essential procedure for improved dentistry due to the additional benefits of deeper healing of the restorative composite and enhanced chemical transformation [16].

The intent is to compare the shear bond strength (SBS) of the composite resin material to that of the conventional luting agents preferred for adhesive restorations after its flow through warm composite has been reduced.

## Materials and methods

Forty-eight discs of lithium disilicate (8 mm in diameter and 3 mm in thickness) were collected and randomly split into four groups of 12. The discs were put in acrylic blocks with an end exposed for bonding purposes after being sliced from lithium disilicate ingots (IPS e.max CAD, Ivoclar Vivadent; Schaan, Liechtenstein), purified, and sintered at 1500 °C for 90 min to 8 mm diameter and 3 mm thickness. Samples were wet ground with a 200-grit silicone carbide abrasive, cleaned in a distilled water-filled ultrasonic bath (Quantrex® 90), and then allowed to air dry. All the disc specimens were etched for 20 seconds with 5% HF (hydrofluoric acid gel) (IPS Ceramic Etching Gel, Ivoclar Vivadent) to strengthen the bond between the cement and the specimen.

The minimum number of samples needed with 80% power, an effect size of 0.50 and 5% significance level was calculated to be 12. This was done using GPOWER software (v 3.0.10; Franz Faul, Kiel University, Kiel, Germany). Our sample comprised 48 samples because we needed to draw comparisons between four groups.

The computerized wax melter's wax will be swapped out for table salt. The wax melter features temperature controls that can be adjusted as needed by the clinician. For composite resin, a wax melter is utilized with a temperature range of 55-65°C for melting the resin to a flowable type; preheating the unit takes 10 minutes, and warming the composite takes two to three minutes. Cements adhere to the surfaces of ceramic specimens (lithium disilicate discs) using polyethylene tubes (PTFE; DuPont Corp.) with an inner diameter and height of 5 and 3 mm, respectively. Materials such as nanohybrid composite resins, resin-modified resin cement, resin cement, and light-activated resin cement are poured into the test tubes, which are then positioned in the center of the specimens' test surfaces. A flat glass was placed on top of the filled polyethylene tubes, and the cement mixture was poured in. The woodpecker (L15000) LED curing unit was used to light-cure the dual-cure resin cement samples for 20 seconds on each side. 

Shear bond strength (SBS) was evaluated with Instron universal testing equipment (Instron 3400, Norwood, MA) at a cross-head speed of 1 mm/min. Once the specimen is securely fastened to the universal testing machine, a knife-edge chisel is employed at a crosshead speed of 1 mm/min to chip away at it until the material cracks or breaks. The resulting load values (N) was changed to the megapascal (MPa) by dividing the failure load (N) by the bonding area (mm^2^).

The study was conducted at the Mahe Institute of Dental Sciences & Hospitals, Chalakkara, Mahe. Ethical Committee clearance was acquired from the Institutional Ethical Committee with approval number MINDS/PG-ETHICAL/008/2020-21. The received details were entered into Microsoft Excel (Microsoft Corp., New York) and analyzed using Statistical Package for Social Sciences (SPSS) (Version 22.0, SPSS, Inc., Chicago, IL). The mean values were evaluated by the Kolmogorov-Smirnov and Shapiro-Wilk tests; the mean between groups using the one-way ANOVA test, and multiple pairwise comparison was done by Tukey's post-hoc test. The criterion of significance was established at a p-value <0.001. While the qualitative data employed proportions, the quantitative data used mean and standard deviation to communicate statistical information.

## Results

The specimens were placed into a universal testing device, and continuous shear bond loads were applied with a knife-edge chisel at a crosshead speed of 1 mm/min at the cement-ceramic interface. As fracture or debonding happens, the test is ended and the highest value is recorded. According to Table 1, the shear bond strength values are normally distributed throughout the four groups. It also shows that p-values are larger than 0.05, indicating statistical insignificance.

**Table 1:** Tests of normality. Df: degree of freedom, GIC: glass ionomer cement. **p-value ≤ 0.001 statistically highly significant, *p-value ≤ 0.05 statistically significant.

Parameter	Groups	Kolmogorov-Smirnov			Shapiro-Wilk		
		Statistics	df	p-value	Statistics	df	p-value
Shear bond strength	Nanohybrid composite resin	0.192	12	0.20	0.86	12	0.09
	Resin modified GIC	0.177	12	0.20	0.89	12	0.12
	Self-adhesive resin cement	0.181	12	0.20	0.95	12	0.64
	Light-activated resin cement	0.163	12	0.20	0.957	12	0.73

The mean value for the nanohybrid composite resin group was 2.2851 ± 0.5901, the resin-modified GIC group was 3.3252 ± 0.7348, the self-adhesive resin cement group was 7.3740 ± 0.6969, and for light-activated resin cement group was 4.4647 ± 0.9774. This result shows statistically high significance with p<0.001 (Table 2).

**Table 2:** One-way ANOVA test. GIC: glass ionomer cement. **p-value ≤ 0.001 statistically highly significant, *p-value ≤ 0.05 statistically significant.

Groups	N	Mean (Mpa)	SD (Mpa)	Min	Max	p-value
Nanohybrid composite resin	12	2.2851	0.5901	1.508	3.818	<0.001**
Resin-modified GIC	12	3.3252	0.7348	1.527	4.162	
Self-adhesive resin cement	12	7.3740	0.6969	6.109	8.391	
Light-activated resin cement	12	4.4647	0.9774	3.152	6.363	

In a series of multiple comparisons, we found that the self-adhesive group had the highest mean compared with the nanohybrid composite resin, resin-modified GIC, and light-activated resin cement groups (p<0.001). It was found that there were significantly higher values in the light-activated resin cement group compared to the nanohybrid composite resin group and the resin-modified GIC (p<0.001 and p=0.004). The mean of p=0.009 was noticed among the resin-modified GIC group and the nanohybrid composite resin group (Table 3).

**Table 3:** Tukey's post-hoc test. GIC: glass ionomer cement. **p-value ≤ 0.001 statistically highly significant, *p-value ≤ 0.05 statistically significant.

Groups (I)	Groups (J)	Mean diff. (I-J) (MPa)	95% CI for the diff.		p-value
			Lower	Upper	
Nanohybrid composite resin	Resin-modified GIC	–1.040	–1.872	–0.208	0.009*
	Self-adhesive resin cement	–5.089	–5.921	–4.257	<0.001**
	Light-activated resin cement	–2.180	–3.011	–1.348	<0.001**
Resin-modified GIC	Self-adhesive resin cement	–4.049	–4.881	–3.217	<0.001**
	Light-activated resin cement	–1.140	–1.971	–0.308	0.004**
Self-adhesive resin cement	Light-activated resin cement	2.909	2.078	3.741	<0.001**

This summarizes that bond strength is significantly high in the self-adhesive resin cement group, followed by the light-activated resin cement group, resin-modified GIC, and least with the nanohybrid composite resin group.

## Discussion

Dental experts have opted to use all ceramics in recent years due to increasing esthetic expectations. Leucite, lithium disilicate, zirconia, and alumina-reinforced ceramics have replaced metallic frameworks in a variety of clinical settings as a result of their invention. These ceramics have superior flexural and compressive strength [16]. This research set out to evaluate how heated composite resin stacked up against more traditional luting cements when it came to shear bond strength.

This research demonstrated that multiple commercial cement solutions obtained widely varying degrees of adhesion to a lithium disilicate substrate. Shear bond strength was found to be greatest for the Rely X U200 and Rely X Veneer cement groups, which used airborne particle abrasion and 5% HF acid etching to improve adhesion to lithium disilicate substrates. Consistent with the present findings, Compton et al. [17] found that light-cured GIC had a greater shear bond strength than chemically-cured. In this research, preheating resin at high temperatures improves its physical properties. The increased radical mobility and subsequent polymerization may account for these findings when the composite is heated [18]. By boosting molecular mobility, preheating the resins to 60°C facilitated greater monomer conversion. Greater crosslinking occurs in compounds with higher conversion, which decreases the free space and enhances their mechanical characteristics [19]. Composite resin flow can be improved by as much as 68% by heating it with a Calset^TM^ composite warmer (Ad Dent, Inc., Danbury, CT, USA). Increasing the flowability of a composite resin can improve its adaptability to the tooth and reduce microleakage [20]. Since composite is a viscoelastic substance, its viscosity and hence its flowability may decrease as its temperature rises [21]. Although heating a composite resin makes it easier to work with, the flow rate differs with composite brands and categories; a pre-heated composite still can't match the workability of a fully heated composite [22]. In accordance with the present study's findings, preheating composites may enhance their physical properties. As the temperature rises, resistance to the flow of composite resins decreases, resulting in effortless adaptability to cavity walls [23,24]. Additionally, several of the physical attributes [25,26] are enhanced, including a higher conversion rate [27] and a lower polymerization shrinkage [28]. Pre-heated composites experience a change in volume and a release of internal stress, but the impact of temperature on these and other qualities needs more research.

The study has some limitations, which include a very small sample size of 48 lithium disilicate discs. A larger sample size might increase the findings' generalizability to a wider population. Lithium disilicate discs (IPS e.max) were used in the study as the testing substrate. Different substrates or restorative materials may interact differently with the luting agents, which could have an impact on the shear bond strength results. Because the investigation was carried out in vitro, the clinical complexity of intra-oral circumstances may not have been accurately replicated. The absence of long-term evaluations that would have allowed for the stability and endurance of the bonds to be observed over protracted times may have prevented the discovery of information about the materials' performance over time.

## Conclusions

The shear bond strength of the self-adhesive resin was shown to be greater using lithium disilicate components in in vitro testing. Although pre-heating composite resins for luting operations can be utilized to decrease the material's viscosity and enhance the restoration setting, it may not increase bond strength.

The results revealed that total etch resin cements are the most effective luting agents and come highly recommended by clinicians for luting lithium disilicate ceramic to dental substrates. Although self-etch and self-adhesive resin cement systems are also widely used, luting lithium disilicate or other all-ceramic restorations is generally regarded as the gold standard. Promptness and tutoring are required for retaining heat for the repair process, including the management of temperature conditions, to ensure successful outcomes. Although choosing resin cement is a matter of clinical circumstance and personal choice, more studies must be done to evaluate its reliability and effectiveness in practice.
